# A rare case of arterial avulsion presenting with occult blood loss following total hip arthroplasty: a case report

**DOI:** 10.1186/1752-1947-3-9320

**Published:** 2009-12-06

**Authors:** Claire Hall, Wasim S Khan, Sohail I Ahmed, David H Sochart

**Affiliations:** 1 Department of Trauma & Orthopaedics, North Manchester General Hospital, Crumpsall Street, Manchester, M8 5RB, UK; 2 Faculty of Life Sciences, University of Manchester, Oxford Road, Manchester, M13 9PT, UK

## Abstract

**Introduction:**

Iatrogenic arterial damage during total hip replacement is a rare but potentially life- or limb-threatening complication. To the best of our knowledge, this is the first reported case of an avulsion injury to a posterior branch of the profunda femoral artery during primary hip arthroplasty.

**Case presentation:**

We describe the case of a 55-year-old Caucasian man who underwent a total hip replacement. The patient's hemoglobin levels dropped postoperatively, but there was no obvious bleeding, hemodynamic instability, pulsatile mass, or limb ischemia. The patient's hemoglobin levels continued to drop despite nine units of transfused blood. Three days after surgery, the patient underwent an angiography that showed an avulsion injury to a posterior branch of the profunda femoral artery. The avulsion was ligated and the hematoma was evacuated.

**Conclusion:**

Vascular damage may present in many ways including obvious bleeding, haemodynamic instability, a pulsatile mass, limb ischemia, and occult blood loss. Any of these signs in isolation or in combination could represent a vascular injury and an urgent angiogram should be considered.

## Introduction

According to the National Joint Registry 2006 Annual Report, over 60,000 primary and revision hip arthroplasties were performed in England and Wales. Complications occurred in around 5% of reported cases of hip arthroplasty. Iatrogenic arterial damage during total hip replacement (THR) is a rare but potentially life- or limb-threatening complication. To the best of our knowledge, this is the first reported case of an avulsion injury to a posterior branch of the profunda femoral artery during primary hip arthroplasty.

## Case presentation

A 55-year-old Caucasian man was admitted for a left-sided metal-on-metal primary hip arthroplasty for osteoarthritis. There was nothing significant in his medical history and he was a non-smoker with a body mass index (BMI) of 38. The patient experienced no significant intra-operative complications but required four units of blood immediately after his operation, which was attributed to intra-operative bleeding.

On the first postoperative day, the patient's hemoglobin level was 65 gm/l. He was subsequently transfused with three units of blood. His low hemoglobin levels were again attributed to intra-operative bleeding. On the second postoperative day, the patient complained of intermittent pain in his left thigh that was associated with numbness and tingling in his toes. His thigh was swollen, but the incision site was dry and there was no pulsatile mass. He remained hemodynamically stable and had distal pulses. His hemoglobin was still only 70 gm/l despite him being transfused with seven units of blood. His clotting profile was also normal. It was thought that the patient had a hematoma which could be was responsible for the pain, the altered sensation, the swelling, and the drop in hemoglobin. The patient was transfused with two more units of blood and four units of fresh frozen plasma. On the third postoperative day, since the patient's hemoglobin remained low despite repeated transfusions, an emergency angiogram was performed.

**Figure 1:**
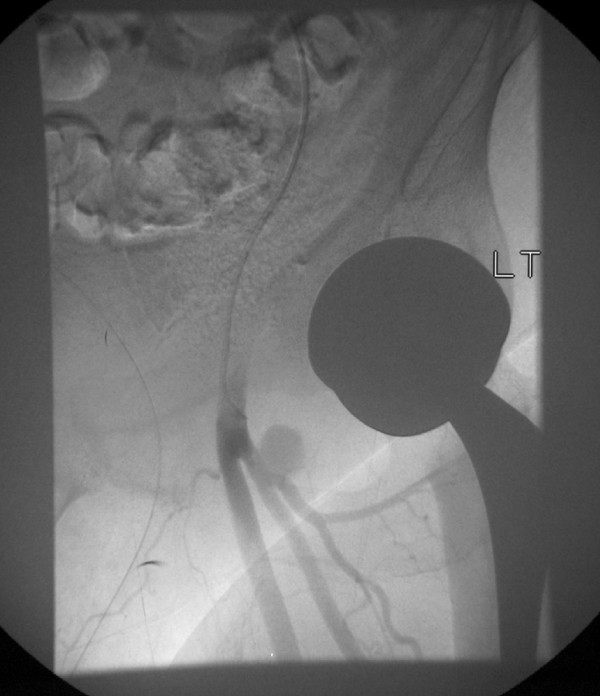
**An angiogram showing an injury distal to the left common femoral artery close to its bifurcation, and adjacent to the profunda femoral artery**.

The angiogram revealed an injury that was distal to the left common femoral artery (Figure 1). The profunda femoral artery was noted to be normal. The vessel was not suitable for embolisation due to its close proximity to the common femoral artery bifurcation. The patient therefore underwent an open exploration of his left groin. An avulsion injury to a posterior perforating branch of the profunda femoral artery was identified and ligated. A left psoas hematoma and a hematoma in the deep tissue of the upper thigh were also drained. The patient recovered well after the second operation and was discharged 12 days after his initial surgery.

## Discussion

The risk of vascular damage after hip arthroplasties is rare and has occurred in only about 0.2% to 0.3% of examined cases [1]. The mechanisms of damage include lacerations, entry of bone cement through a defective acetabulum, development of pseudoaneurysms, and arteriovenous fistulas [2]. There are some reports that lacerations of the common femoral artery and its branches commonly occur after hip fractures are repaired. The arterial damage occurs as a result of a tear from an avulsed bone fragment [3-6], or when surgical instruments such as retractors or protruding screws damage the artery [6,7].

Vascular damage secondary to contact with cement may occur when the cement leaks through a defective acetabulum, as in cases where over-reaming occurs [1]. Early complications include thrombotic occlusion as a result of the exothermic reaction produced when cement hardens [7]. Later complications occur if the vessel becomes adherent to the bone or the prosthesis itself. Later dislocation or revision of prostheses results in lacerations or avulsions of the blood vessel.

Aneurysms are localised dilatations of the blood vessels. True aneurysms are bounded by all the layers of the blood vessel, whereas false or pseudoaneurysms are extravascular hematomas. Atherosclerosis is the most frequent cause of aneurysm as it causes the thinning of the arterial wall. This process also makes iatrogenic damage more likely as the diseased vessel can no longer withstand normal stresses [2]. Retraction is necessary to prepare both the acetabulum and the femoral head before implant insertion. Hohmann retractors have been implicated in several cases of intra-operative vascular injury [2,6-8]. Both blunt- and sharp-ended retractors have caused lacerations and pseudoaneurysms.

Injuries to the external iliac, common femoral, profunda femoral, medial femoral circumflex and lateral femoral circumflex arteries during hip operations have been reported [2]. Profunda femoral artery damage is very rare as the artery is not close to the operation site, but considerable anatomical variation can occur. The profunda femoral artery originates from the common femoral artery at 0 cm to 8 cm below the midpoint of the inguinal ligament in the medial thigh [9]. The most common origin is posterolaterally, but the artery may originate posteriorly or posteromedially. The medial and lateral circumflex arteries arise from the profunda femoris along with three to six perforating branches at variable locations [9]. Wilson *et al. *described one case of profunda femoral artery damage in over 4,000 elective procedures [10].

In the case of our patient, the avulsed vessel is unlikely to have been damaged directly due to its location in the medial thigh. It is possible that the vessel was avulsed when the hip was manipulated during operation, since the hip was flexed and forced into internal rotation and adduction to dislocate it prior to preparing the acetabulum and the femur. The patient was particularly large and muscular and the force required to dislocate his hip may have put excessive traction on the vessel. The patient's large size also contributed to the delay in the postoperative diagnosis of hematoma. Although the presence of atherosclerosis would make this type of injury more likely, the patient had no pre-existing vascular disease and the arteries looked entirely normal without any atherosclerotic changes on the angiogram (Figure 1).

## Conclusion

Iatrogenic arterial damage during THR is a rare but potentially life- or limb-threatening complication. Avulsion injuries and injuries to the profunda femoral artery or its branches may be uncommon, but they can still occur. Vascular damage may present in many ways including obvious bleeding, hemodynamic instability, a pulsatile mass, and limb ischemia. Any of these signs in isolation or in combination could represent a vascular injury, hence an urgent angiogram should be considered. Particular attention should be given to those with a BMI of over 30 or to those who are known to have pre-existing atherosclerotic disease as these factors could make certain types of injury more likely to occur.

## Abbreviations

BMI: body mass index; THR: total hip replacement.

## Consent

Written informed consent was obtained from the patient for publication of this case report and any accompanying images. A copy of the written consent is available for review by the Editor-in-Chief of this journal.

## Competing interests

The authors declare that they have no competing interests.

## Authors' contributions

CH collected, analyzed and interpreted patient's data, and also researched the related literature. SA prepared the final drafts of the manuscript. WK and DS supervised CH and SA, and were major contributors in writing the manuscript. All authors read and approved the final manuscript.

## References

[B1] Bechet FR Himmer O Mairy Y Lootvoet L Arterial false aneurysm after total hip arthroplasty. A case report Rev Chir Orth Reparatrice Appar Met 2004 90 365 368 10.1016/s0035-1040(04)70133-6 15211266

[B2] Aust JC Brendenburg CE Murray DG Mechanisms of arterial injury associated with total hip replacement Arch Surg 1981 116 345 349 7469777 10.1001/archsurg.1981.01380150063017

[B3] Ha YC Luminita S Cho SH Choi JY Koo KH Laceration of femoral vessels by an avulsion fracture fragment of the lesser trochanter after bipolar hemiarthroplasty J Arthroplasty 2005 20 680 683 10.1016/j.arth.2005.04.005 16310008

[B4] Mauerhan DR Maurer RC Effeney D Profunda femoris arterial laceration secondary to intertrochanteric hip fracture: a case report Clin Orthop Relat Res 1981 161 215 219 7307383

[B5] Obry C Mertl P Woestelandt T Vives P False aneurysm of the profunda femoris artery after fracture of the upper end of the femur. Apropos of a case Rev Chir Orthop Reparatrice Appar Met 1988 74 585 587 3070654

[B6] Karanikas I Lazandes M Arvanitis D Papayanopoulos G Exarchou E Dayantas J Iatrogenic arterial trauma associated with hip fracture surgery Acta Chir Belg 1993 93 284 286 8140841

[B7] Nachbur B Meyer RP Verkkala K Zürcher R Mechanisms of severe arterial injury of the hip Clin Orthop Rel Res 1979 144 122 133 477093

[B8] Bansal R Laing PW Iatrogenic blunt arterial injury during hip fracture surgery Acta Orthop Belg 2006 72 96 99 16570904

[B9] Siddharth P Smith NL Mason RA Giron F Variational anatomy of the deep femoral artery Anat Rec 1985 212 206 209 10.1002/ar.1092120216 3842043

[B10] Wilson JS Miranda A Johnson BL Shames ML Back MR Bandyk DF Vascular injuries associated with elective orthopaedic procedures Ann Vasc Surg 2003 17 641 644 10.1007/s10016-003-0074-2 14534848

